# Improving the Mould and Blue-Stain-Resistance of Bamboo through Acidic Hydrolysis

**DOI:** 10.3390/polym14020244

**Published:** 2022-01-07

**Authors:** Zixuan Yu, Xiaofeng Zhang, Rong Zhang, Yan Yu, Fengbo Sun

**Affiliations:** 1 Department of Biomaterials, International Center for Bamboo and Rattan, Beijing 100102, China; yuzixuan@icbr.ac.cn (Z.Y.); zhangxf@icbr.ac.cn (X.Z.); zhangrong@icbr.ac.cn (R.Z.); 2 SFA and Beijing Co-Built Key Laboratory of Bamboo and Rattan Science & Technology, State Forestry and Grassland Administration, Beijing 100102, China; 3 College of Material Engineering, Fujian Agriculture and Forestry University, Fuzhou 350002, China; yuyan9812@outlook.com

**Keywords:** acidic treatment, bamboo, colour, mould-resistant, starch

## Abstract

Bamboo is much more easily attacked by fungus compared with wood, resulting in shorter service life and higher loss in storage and transportation. It has been long accepted that the high content of starch and sugars in bamboo is mainly responsible for its low mould resistance. In this paper, acetic acid, propionic acid, oxalic acid, citric acid, and hydrochloric acid were adopted to hydrothermally hydrolyze the starch in bamboo, with the aims to investigate their respective effect on the mould and blue-stain resistance of bamboo, and the optimized citric acid in different concentrations were studied. The starch content, glucose yields, weight loss, and colour changes of solid bamboo caused by the different acidic hydrolysis were also compared. The results indicated that weak acidic hydrolysis treatment was capable of improving mould-resistant of bamboo. The mould resistance increased with the increased concentration of citric acid. Bamboo treated with citric acid in the concentration of 10% could reduce the infected area ranging to 10–17%, the growth rating of which could reach 1 resistance. The content of soluble sugar and starch remained in bamboo decreased significantly from 43 mg/g to 31 mg/g and 46 mg/g to 23 mg/g, respectively, when the citric acid concentration varied from 4% to 10%. Citric acid treatments of 10% also caused a greatest surface colour change and weight loss. The results in this study demonstrated citric acid treatment can effectively reduce the starch grain and soluble sugars content and improve mould resistance of bamboo, which can be attributed to the reduction of starch grain and soluble carbohydrates (such as glucose, fructose, and sucrose, etc.) in bamboo.

## 1. Introduction

Bamboo is easily attacked by various fungi, among which the infection caused by mould fungi is the most common and serious. Mould fungi can infect bamboo during storage, transportation, processing, and utilizing, with notable appearance, and in some cases, it can cause illness to humans. This greatly limits the application of solid bamboo and bamboo-derived products. The low mould resistance of bamboo is mainly attributed to its high content of starch and free sugars, which can act as feed for fungus or insects [1,2].

Many inorganic and organic preservatives, such as CCA, ACQ, CCC, DDAC, and IPBC, demonstrate a certain degree of mould resistance. Nevertheless, few of them perform well as a bamboo-mould inhibiter [3]. This is because the majority of them were actually developed for wood materials. Furthermore, the anatomic characteristics and chemical compositions of bamboo are quite different from wood [4]. There is no transverse conductive tissues in bamboo, which makes bamboo inaccessible to many preservatives with high molecular weight. Furthermore, the wide applications of these preservatives might lead to major environmental concerns [5,6,7]. Recently, wood furfurylation was found to be a highly efficient approach to deal with the mould issue on wood [8], but the colour of the treated wood was typically turned into black or brown, losing its original natural appearance.

Since the existence of starch and free sugars in bamboo is directly related to its mould deterioration, a reasonable way to improve the durability of bamboo is to remove these nutritious components from bamboo. The starch in bamboo is mainly located in the cavities of its ground parenchyma cells with normally less than 6% of total bamboo mass. The plant starch is stored in crystalline form and is nearly insoluble in water at ambient temperatures [9,10]. However, it can be hydrolysed to sugars in an acidic solution or enzyme catalyst. Clausen [11] pointed that multifactorial fatty acid emulsifications incorporated as an appropriate adjuvant are effective in inhibiting mould on wood products. Organic acids, such as boric, citric, and sorbic acids, have long been used in the food industry as preservatives [12,13]. Tang [14,15] found that bamboo dipped in 7% propionic acid or 10% acetic acid were effective in inhibiting mould growth completely. However, their effectiveness was mainly attributed to their acidity. Other monocotyledons, like palm woods, which also contain much starch and soluble carbohydrates, were also prevented from fungal colonization by acetic and propionic acid [16]. Sun also found that 1% HCl was helpful to improve mould resistance of bamboo [17], but the supplementary tests with water immersing the treated specimens performed significant resistance as well. The results indicated that acidic treatments are promising against fungus-affected bamboo. However, the differences of acid types in inhibiting mould and their mechanisms are still not identified completely.

In this study, four kinds of low molecular weight organic acids, namely acetic acid, propionic acid, oxalic acid, and citric acid, as well as one inorganic acid, namely hydrochloric acid, were adopted to hydrothermally hydrolyze the starch in bamboo. The mould resistance, weight loss, and colour changes of solid bamboo as well as the glucose yields in the respective hydrolysates caused by the different acidic hydrolysis were compared. The objective of this study is to evaluate the significance of starch in bamboo for mould growth and whether acidic treatment can achieve satisfactory mould resistance for bamboo.

## 2. Material and Methods

### 2.1. Materials

Moso bamboo (*Phyllostachys pubescens* Mazel ex H. de Lehaie) strips with regular cross section were purchased from Hangzhou Dazhuang Flooring Co., Ltd. (Dazhuang Flooring Co., Ltd., Hangzhou, China). The samples for mould resistance test had dimensions of 50 mm (longitudinal) × 20 mm (tangential) × 5 mm (radial), whereas the ones for both weight loss and colour change measurement were 20 mm (longitudinal) × 20 mm (tangential) × 5 mm (radial). For each treatment, there were 12 specimens for mould resistance tests and 10, respectively, for weight loss and colour change tests. Five kinds of common acids, namely acetic acid, propionic acid, oxalic acid, citric acid, and hydrochloric acid, were purchased from Beijing Chemicals (Beijing, China). The mould fungus (*Aspergillus niger* van. Tieghem) and the blue-staining fungus (*Botryodiplodia theobromae* Pat.) were purchased from the Institute of Forest Ecology Environment and Protection, Chinese Academy of Forestry (Beijing, China). The mould fungus of *Penicillium citrinum* and *Trichoderma viride* were purchased from Institute of Microbiology, Chinese Academy of Sciences (Beijing, China).

### 2.2. Acidic Treatment

Bamboo specimens were soaked in the aqueous solutions of acetic acid, propionic acid, oxalic acid, citric acid, and hydrochloric acid with concentration (W/W) of 2%, 2%, 2%, 2%, and 0.7%, respectively, for 1 h at room temperature. Then, the samples together with the acidic solutions were transferred to a drying oven and heated at a temperature of 90 °C for 3 h. Afterwards, the residual acids in the treated samples were removed by repetitive washing with deionized water. All samples were then oven dried at 105 °C. Bamboo specimens treated with citric acid of 4%, 6%, 8%, and 10% concentration were soaked for 1 h at room temperature as well and then oven-dried at 90 °C for 2.5 h, then washed with deionized water and oven dried at 105 °C.

### 2.3. Mould Resistance Tests

The tests of laboratory mould resistance were carried out according to a Chinese national standard GB/T 18261-2000. The treated and control (untreated) blocks were placed in petri dishes containing agar (2% agar) with the selected fungus and incubated for 4 weeks at 25 °C and 85% relative humidity. The change of each sample was photographed and recorded every day. The samples were visually rated for the growth of fungi on the following scale: 0 = no growth, 1 = 25 percent, 2 = 50 percent, 3 = 75 percent, and 4 = 100 percent coverage with mould. To evaluate growth of fungi objectively, the infection area on bamboo surface after the test was estimated by Matlab (MathWorks, America, R2011b). The infection degree of bamboo was obtained from ratio of hypha pixels to the whole block pixels.

### 2.4. Soluble Sugar Content in the Hydrolysates

Ion chromatography with an ampere detector (850, Metrohm, Switzerland) was used to test the content of glucose in the hydrolysates. Solutions after acidic treatments at 90 °C were collected and centrifuged, and the supernatant fluid was recovered and injected into the centrifuge tube for analysis. The injection volume was 10 mL. The ion chromatographic analysis by using a Hamilton RCX-30 aminex was performed at 20 °C. The ultrapure water solution was 2.0 mM NaOH + 0.5 mM NaAc. The flow rate of the eluent was 1.0 mL/min and kept for 60 min.

High-performance liquid chromatography (Waters Alliance e2695, Waters Co., Milford, MA, USA) was also used to test soluble sugar content in the hydrolysates of citric acid treatments. The working condition of HPLC was Bio-Rad87-H column, column temperature of 65 °C, and sulfuric acid as mobile phase. The flow rate was 0.6 mL/min.

### 2.5. Soluble Sugars and Starch Content Remained in Bamboo

Residual soluble sugars content of bamboo was determined based on the method of anthrone colorimetry. The main procedure was as follows: 0.1 g of bamboo powders were ground by pestle by using 80% alcohol and then heated in water baths at 80 °C for 30 min. The supernatants collected by centrifugation were transferred in a 100-mL volumetric flask after three replications. Next, 4 mL of anthrone solution was added to the diluted solution in the ice water bath and shaken well. Next, we boiled the tube in a boiling water bath for 10 min. The absorbance at 630 nm was read. 

Residual starch of bamboo was determined using the method of anthrone colorimetry too. The residues after extraction were dried at 80 °C and thereafter boiled with deionized water for 15 min. We next put the centrifugal tube into an ice water bath, then added 2 mL perchloric acid (9.2 mol/L) for 15 min, and centrifuged it for supernatant. Next, 4.6 mol/L perchloric acid were added to the sediment and extract for 15 min to obtain the supernatant. Then, we washed the sediments with 7 mL distilled water and performed a water bath for 20 min, and then, the supernatants were transferred to the volumetric bottle. We then took 2 mL solutions in the tube and added them into 4 mL anthrone and boiled them for 7.5 min. The absorbance at 630 nm was determined. 

### 2.6. Color Change 

The colour changes of specimens due to acidic treatments were measured by a portable chromatic aberration meter (BYK Gardner-6834, Geretsried, Germany). The value of CIE L*, a*, and b* colour parameters of the samples were obtained directly from the meter. Color change was evaluated according to the ISO 7724 standard. The overall colour change (∆E*) of the treated samples was calculated according to the following equation:

(1) $$\Delta E*=\sqrt{{\left(\Delta L*\right)}^{2}+{\left(\Delta a*\right)}^{2}+{\left(\Delta b*\right)}^{2}}$$

where ∆L*, ∆a*, and ∆b* represent the changes in L*, a*, and b* of samples after acidic treatment, respectively. Ten replicates were tested, and the average value was calculated for colour analysis.

### 2.7. Weight Loss Ratio

Weight loss ratio of samples was calculated according to Equation (2):

(2) $$WL=\frac{W_{0}-W_{1}}{W_{0}}\times 100\%$$

where WL is weight loss ratio of each sample (%); W_0_ and W_1_ represent the oven dried weight of samples before and after acidic treatment, respectively.

### 2.8. SEM Bbservation 

An Environmental Scanning Electron Microscopy (ESEM XL 30, FEI, Hillsboro, OR, USA) was used to compare the microstructure of bamboo before and after acidic treatment. The acceleration voltage was set at 7–10 kV.

## 3. Results and Discussion

### 3.1. Treatments with Acids Concentration below 2%

#### 3.1.1. Mould-Resistance Test

Bamboo infected by *A. niger* and *B. theobromae,* respectively, were observed periodically. The occurrence and propagation of both fungi on the treated samples were all much later and slower than those on the control samples. This phenomenon was especially obvious during the period of the first 3–5 days. For example, *A. niger* appeared on the control bamboo strips as early as the third day and then spread over the whole surface in less than five days. In contrast, the occurrence of mycelia on the treated bamboo was postponed by 2–7 days, dependent on the acid used. Furthermore, the treatments with oxalic acid, citric acid, and hydrochloric acid showed better fungus resistance performance than the other two acids, as the date of first occurrence was postponed, and the mycelia on the surfaces were much thinner during the first week. During the second week, the propagation of fungi started to speed up, but it did not cover the whole surface yet. Until the fourth week, all the treated samples were seriously infected by the fungi, with mycelia spreading over the surface. The growth rating of for treated groups ranged between 2 and 3 compared to 4 for the control group (Table 1). The results showed that although the distribution density and growth rate of mycelia and spores on the surface of treated bamboo were much lower than that of untreated samples, significant differences were observed for 2% organic acidic treatments in terms of the infected areas ratio at the final date of a standard mould-resistance experiment. For *B. theobromae*, the visible blue stain happened during the second week. The growth rating ranged between 2 and 3 for treated groups and up to 4 for control group.

**Figure 1:**
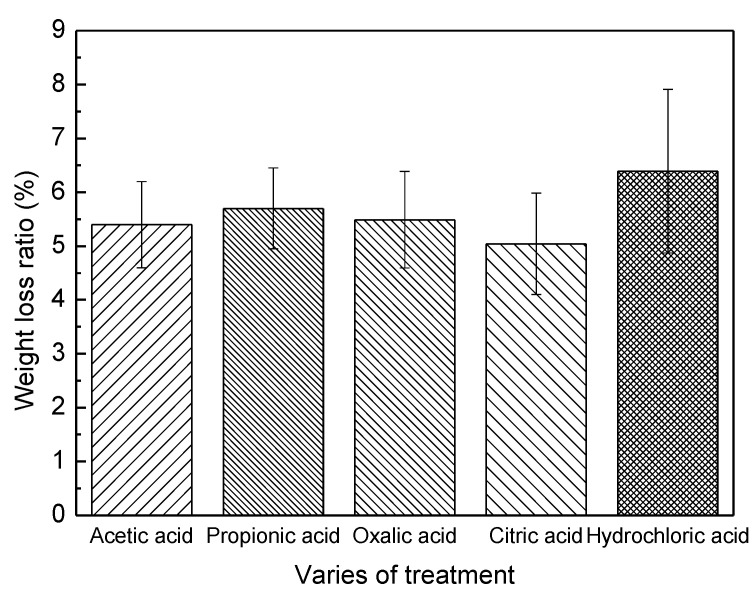
Weight loss of bamboo caused by five different acidic treatments.

The general improvement in mould and blue-stain resistance of bamboo after acidic treatments could be to a large extent attributed to the reduction of nutritious components in bamboo, including free sugars and starch particles. Previous studies [2,18,19] have indicated that the presence of considerable quantities of starch and sugars in bamboo make it more attractive to organisms, especially mould and stain fungi and borer beetles. Wingfield and Schmidt also pointed out that stain fungi can easily get nutrients from soluble sugars and starch [20,21]. Huang demonstrated that a combination of pressurized hydrothermal treatment and amylase hydrolysis could improve the mould resistance of Moso bamboo due to the reduction of free sugars and starch content [22]. The acidic treatments in the present study follow a similar mechanism. Figure 1 shows a weight loss of 5.04–6.39% of bamboo after various acidic treatments. This indicated that some starch and extractives were removed after hydrothermal acidic treatments, representing a significant reduction in the nutritious contents in bamboo. Another possible reason is that some water-solution-free sugars in bamboo were also washed out during the experiments. Schmit showed that the contents of starch and sugars of palm woods samples were considerably reduced by three days of watering [16]. However, although acidic treatments can improve the fungus resistance of bamboo to a certain extent, it seems that the earlier differences in mould time of the five groups may be more influenced by the remaining nutrients. In addition, the removal of starch and free glucose was incapable of totally solving the problem, as serious mould growth still occurred after one month of incubation. This contrasts to the results on palm woods, where the decrease of starch and sugar contents reduced mould and blue-stain growth [16]. A possible explanation might be related to the acidic decomposition of xylan in bamboo, producing additional monosaccharides or oligosaccharides that instead facilitate the appearance of mildews.

#### 3.1.2. Glucose Yields in Different Hydrolysates

**Figure 2:**
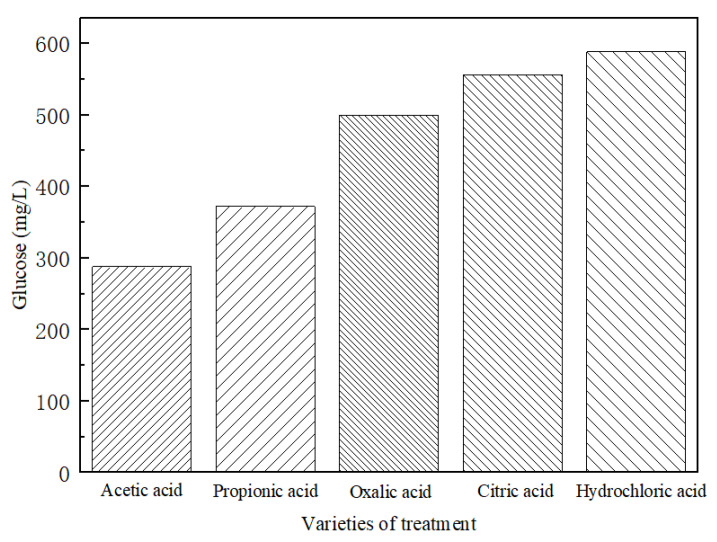
Glucose content in five different acid hydrolysates.

The glucose yields in different hydrolysates are shown in Figure 2. The glucose yields in hydrolysates derived from acetic acid and propionic acid were 288 and 372 mg/L, respectively, significantly lower than the 500, 557, and 588 mg/L, respectively, from oxalic acid, citric acid, and hydrochloric acid. Among the five kinds of acids used in the present study, the inorganic hydrochloric acid showed the highest hydrolysis performances although its concentration in the solution was only 0.7%, significantly lower than 2% for the other four organic acids. In addition, acetic acid and propionic acid were much weaker than oxalic acid and citric acid in hydrolysis because they provided lower glucose yields in the hydrolysates. The acidic hydrolysis of starch is a highly complex process. This process is related to several factors, including hydrolysis time, temperature, acid types and concentrations, etc. During the process of treatment, organic acids may become involved in esterification reactions with starch. However, the key reason for the differences in glucose yields by different organic acids cannot be highly clear without sufficient investigation. In addition, mass loss was mainly influenced by the hydrolysis of bamboo starch and the dissolution of some extractives, but there was no significant difference among the four organic acids. The result indicated that the hydrolysis reaction and other chemical reactions take place when bamboo is treated with the organic acids selected. 

**Figure 3:**
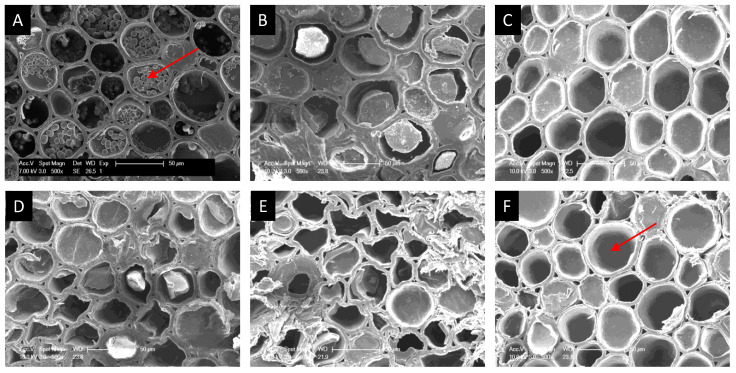
Distribution of starch in bamboo parenchyma cells ((**A**) Control sample (starch grains (red arrow) are rich in the parenchyma cells)): (**B**) acetic acid treatment sample, (**C**) propionic acid treatment sample, (**D**) oxalic acid treatment sample, (**E**) citric acid treatment sample, and (**F**) hydrochloric acid treatment sample (starch grains disappeared (red arrow) in the parenchyma cells).

Several papers [14,23] showed that the prevention of fungal growth on bamboo samples was mainly inhibited by impregnating sample outer layers with acids of low pH value so that fungi could not start their growth. The main chemical components of bamboo culms include cellulose, hemicellulose, and lignin, which account for above 70–90% of total bamboo mass [24,25]. Moreover, there are about 2–6% starch, 2% deoxidized saccharide, 2–4% fat, and 0.8–6% protein in bamboo [26]. Cellulose is highly difficult to be hydrolysed under the present weak acidic solutions. Xylose contains more than 95% of the sugar units in the hemicellulose of Moso bamboo [27]. Therefore, the starch in bamboo was obviously the main glucose source in this study. Starch has been the primary source of various sugars in the industry (such as glucose, maltose, some oligosaccharide, etc.), with the action of acid, for a long time [28,29]. Additionally, the free glucoses in bamboo can be seen as another source of glucoses in the hydrolysate. Figure 3 illustrates the cross-section images of the untreated and acid-treated bamboo. It can be observed that the starch grains in the parenchyma cells disappeared mostly after being treated with propionic acid, oxalic acid, citric acid, and hydrochloric acid. However, this does not mean the whole starch had been hydrolysed into glucose. Some of it could still exist in bamboo but with the new structure or even smaller sizes that were invisible under SEM. 

#### 3.1.3. Color Changes Due to the Treatments

Table 2 shows the colour changes (∆L*, ∆a*, ∆b*, ∆E*) of bamboo due to acidic treatments. The surface colour changed from light to dark, as indicated by a decrease in lightness (L*). Besides, the treated surfaces became reddish and blue with increased a* and reduced b*, respectively. It can also be observed that the overall colour change value (∆E*) increased after acidic treatments. Similar to the tendency of glucose yields with different acidic treatments, the samples treated with hydrochloric acid showed highest ∆E* (6.83), followed by those treated with oxalic acid (5.62), citric acid (5.98), acetic acid (5.15), and propionic acid (4.64). Color changing after different treatments indicates the existence of chemical reactions in the acidic conditions. The different discoloration behaviors of bamboo indicate that the surface colour of bamboo was influenced by acid types, concentration, and pH, etc. It has been reported that the lignin and extractives in bamboo (such as tannins and colour pigments, etc.) can lead to the colour changes of bamboo/wood under the reaction of light and oxygen [30,31,32]. In this study, the colour variations on bamboo surface may be caused by the dissolution or reaction of bamboo extractives with acids and heat as well as minor hydrolysis of hemicelluloses. 

### 3.2. Citric Acid Treatments with Different Concentration

Since the acidic treatments with oxalic acid, citric acid, and hydrochloric acid showed a better fungus resistance performance in the first week, further study should be focused on the mould resistance treated with higher acid concentration, which can make the hydrolysis of starch grains highly effective. Though the oxalic acid and hydrochloric acid are both effective agents for mould and blue-stain resistance, citric acid is superior to all of them considering their environmentally friendly and edibility. Therefore, bamboo treated with citric acid in different concentration was further investigated. 

#### 3.2.1. Mould Resistance Test

Bamboo strips that treated with citric acid from 4–10% concentration were tested in group for their ability against mould and blue stain fungi. The result shows that citric acid treatment was highly effective in refraining bamboo mildew, and the degree of refraining increased with the increased concentration. 

**Figure 4:**
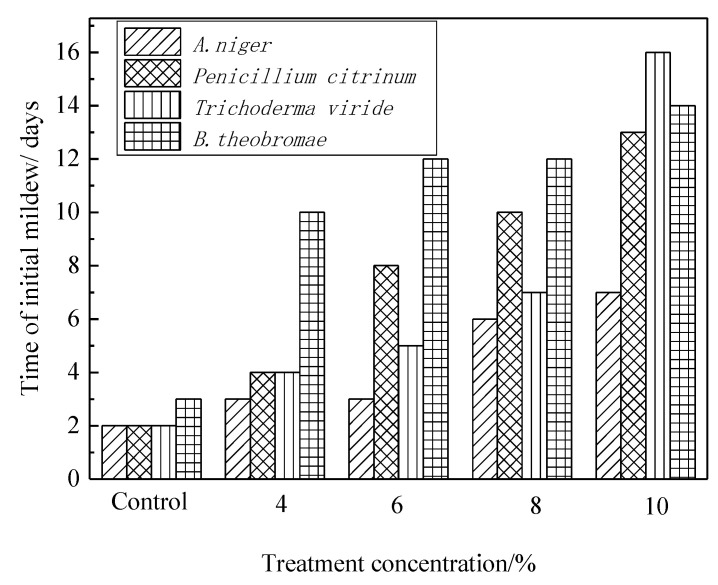
Time of initial mildew for bamboo treatment with different citric acid.

Figure 4 shows that the mycelia and spores of *A. niger* appeared on the surface of bamboo strips on the third day when treated with 4% concentration level of citric acid, whereas the time can be postponed until the seventh day, as long as the concentration of the citric acid is changed to 10%. For the *Penicillium citrinum* and the *Trichoderma viride*, the time of the appearance of mycelia and spores can be postponed from the fourth day to the thirteenth and sixteenth day, respectively, by adjusting the concentration level of the citric acid as well. For *B. theobromae*, the visible blue-stain spots occurrence lasted 14 days. The chart illustrate the significant upward trend in the occurrence of mycelia on the treated bamboo. 

**Figure 5:**
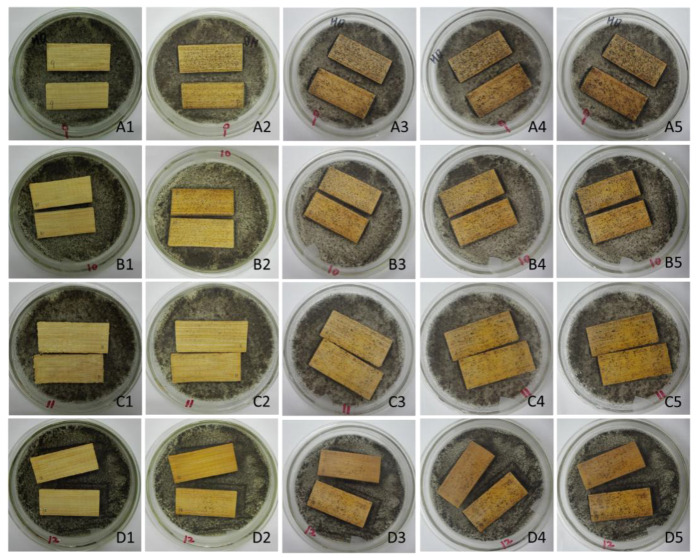
The bamboo samples infected by *A. niger* in different periods (**A1**–**A5** show the mould growth on samples at Day 0, 7, 14, 21, and 30 when treated with 4% citric acid; **B1**–**B5** show the mould growth on samples at Day 0, 7, 14, 21, and 30 when treated with 6% citric acid; **C1**–**C5** show the mould growth on samples at Day 0, 7, 14, 21, and 30 when treated with 4% citric acid; **D1**–**D5** show the mould growth on samples at Day 0, 7, 14, 21, and 30 when treated with 4% citric acid.).

**Figure 6:**
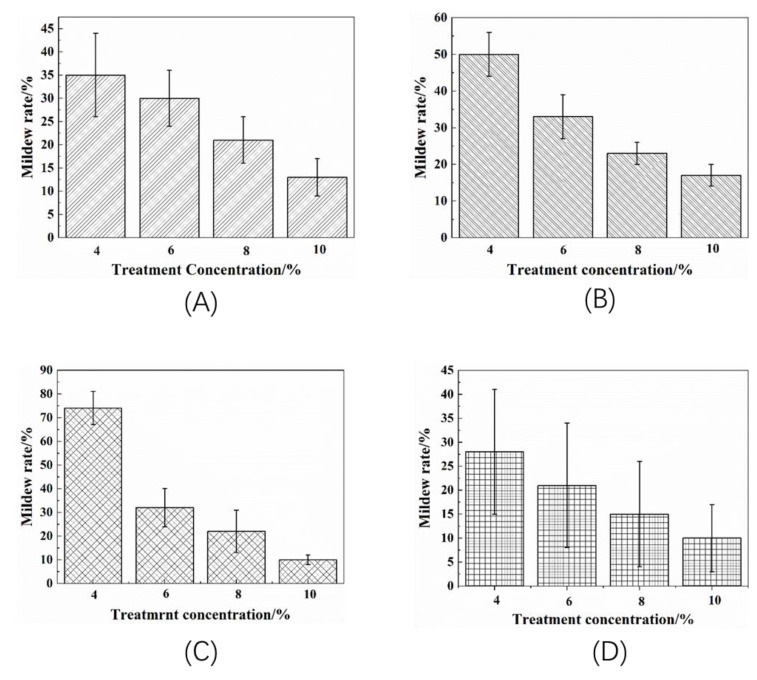
Mildew rate of bamboo in different treatment concentrations (**A**–**D** is for *A. niger*, *Penicillium citrinum*, *Trichoderma viride,* and *B. theobromae*, respectively).

Obviously, the average infected areas for four treated groups decreased with increased concentration of citric acid. As an example, Figure 5 shows the bamboo samples infected by *A. niger* in different periods. As shown in Figure 6, for bamboo strips treated with citric acid concentration ranging from 4% to 10%, the average areas infected by *A. niger*, *Penicillium citrinum*, *Trichoderma viride,* and *B. theobromae* decreased from 35% to 13%, 50% to 17%, 74% to 10%, and 28% to 10%, respectively. Moreover, the mildew performance of the 10% concentration treatment was significantly higher than other three treatments, reaching a range of infected area of 10–17% that was three to eight times that treated with 4% concentration citric acid after four weeks when infected by four different moulds.

From the results of infected areas, it is known that the growth rating can be controlled within 1 resistance by treated with 8% or 10% citric acid. Additionally, for *A. niger*, *Penicillium citrinum,* and *B. theobromae*, the prevention efficiency of bamboo treated with 10% concentration was 75%, while *Trichoderma viride* was slightly lower.

#### 3.2.2. Glucose Yields and Starch Remained

**Figure 7:**
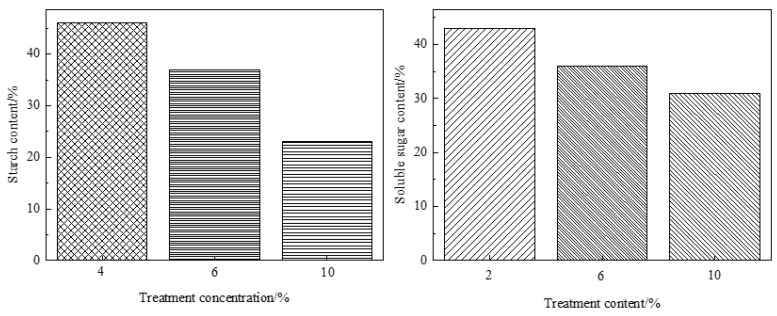
Starch and soluble sugar remaining in bamboo.

To clarify the effect of citric acid concentration on bamboo, soluble sugar and starch remaining in bamboo were tested separately. Both of them showed a downtrend with increased acid concentration (Figure 7). The soluble sugar remaining in bamboo was 43 mg/g, 36 mg/g, and even 31 mg/g when citric acid concentration was 4%, 8%, and 10%, respectively. The content of starch remaining in bamboo was 46 mg/g, 37 mg/g, and even 23 mg/g when citric acid concentration was 4%, 8%, and 10%, respectively. 

**Figure 8:**
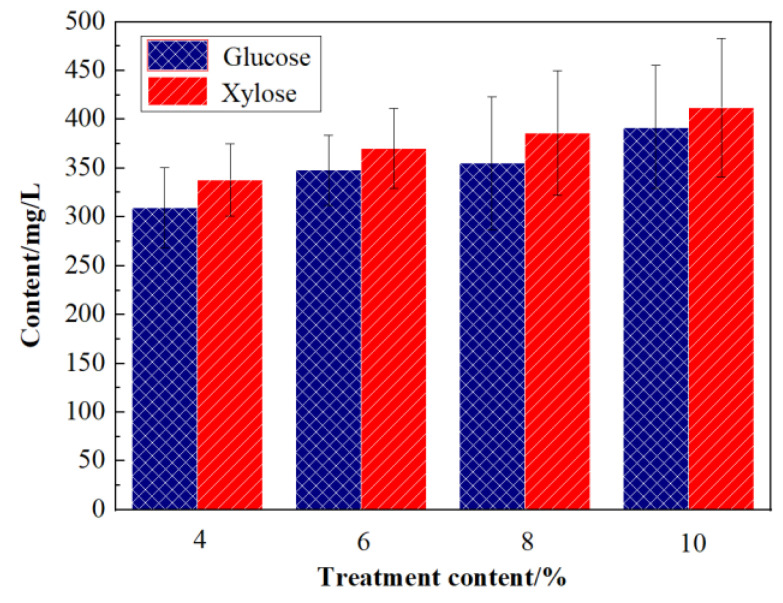
Glucose and xylose contents in different hydrolysates.

**Figure 9:**
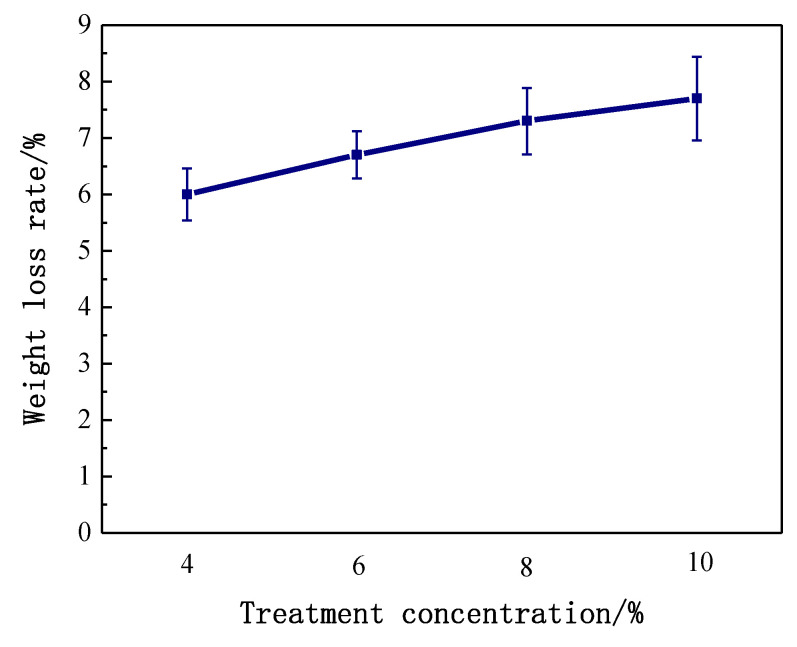
Weight loss of bamboo caused by different citric acidic treatments.

To further clarify the hydrothermal citric acid treatments in different concentration, dissolved glycosyls were tested by analyzing conditioning fluid. The glucose and xylose yields in hydrolysates derived from citric acid are shown in Figure 8. A marked effect was observed in the citric acid of 10%. The increases in glucose and xylose ranged from 310 and 338 mg/L to 392 and 412 mg/L, respectively, indicating acidic hydrolysis of starch increased with increased citric acid concentration. Consistently, the weight loss of treated bamboo increased with increased concentration. It was measured that the weight loss of bamboo was from 6% to 7.6% as citric acid concentration increased from 4% to 10% (Figure 9). 

The weight loss of bamboo and contents of sugars in hydrolysate showed a similar trend. Based on the results of Figure 9, it is clear that citric acid concentration contributed to hydrolysis and dissolution of starch in bamboo. Furthermore, the sugars in the conditioning fluid were mainly produced by citric acid hydrolysis. On the other hand, citric acid has the potential to react with cell wall polymers. Feng [33] pointed out that citric-acid-treated wood exhibited considerable improvements in dimensional stability, as wood and citric acid can react by ester linkages. The improvement of bamboo mould resistance could also be due to esterification between citric acid and bamboo. 

#### 3.2.3. Color Changes Due to the Treatments

Considering the chemical changes brought about by citric acid, especially in its high concentrations, colour changes of treated bamboo in different concentrations were tested. The result shows that the parameter ∆E* of 10% citric acid treatment had the highest value, 5.002. For the gradually increasing discoloration of bamboo, one of the reasons the colour changed on the bamboo surface was due to the dissolution of bamboo extractives; additionally, the high concentrated citric acid reacted readily under heating conditions. The colour change can be attributed to the decomposition of citric acid to unsaturated acids, such as acetone dicarboxylic acid and aconitic acid, when heated at high temperatures. Feng [33] reported that samples treated with citric acid at variable WPG levels caused colour change. For example, at 6% of WPG level, a slight yellowing was visible, and the wood was even darker at higher WPG level.

## 4. Conclusions

Mould resistance of bamboo strips treated with low-molecular-weight organic acids and inorganic acid were first tested, and then effect of citric acid with different concentrations were studied. Bamboo treated with acetic acid, propionic acid, oxalic acid, citric acid, and hydrochloric acid in a low concentration could improve their fungus growth rating from 4 in control samples to 2 or 3 resistance. However, there was no remarkable difference in final inflected areas of bamboo surface among different acidic treatments. Citric acid is effective to refrain mildew, and the mould resistance increased with the increased concentration of citric acid, and the fungus growth rating could reach 1 resistance when citric acid concentration was greater than 8%, while bamboo treated with citric acid in the concentration of 10% could control the infected area in the range of 10–17%. The improved mould and blue-stain resistance of treated bamboo could be attributed to the reduced nutrients in bamboo due to the hydrolysis of starch grains in parenchyma cells and the dissolution of soluble sugar. Compared with the treatments with other acid concentration, 10% citric acid treatments produced the greatest dissolved glycosyls content, colour change, and weight loss value. 

## Figures and Tables

**Table 1:** Fungus growth rating of bamboo treated with different acids.

Varieties of Treatment	*A. niger*	*Penicillium citrinum*	*Trichoderma viride*	*B. theobromae*
Control	4	4	4	4
Acetic acid	3	3	3	3
Propionic acid	3	3	3	3
Oxalic acid	3	3	2	2
Citric acid	3	2	2	2
Hydrochloric acid	2	2	2	2

**Table 2:** Color parameter changes of bamboo due to acid treatments.

Parameter	Acetic Acid	Propionic Acid	Oxalic Acid	Citric Acid	Hydrochloric Acid
∆L*	–4.329	–2.945	–4.897	–4.979	–6.127
∆a*	0.46	0.249	1.557	0.22	2.214
∆b*	–2.747	–3.578	–2.276	–3.304	–2.061
∆E*	5.15	4.64	5.62	5.98	6.83

## Data Availability

The data presented in this research are available on request from the corresponding author.
